# Systematic review: the influence of nasal obstruction on sleep apnea^☆^

**DOI:** 10.1016/j.bjorl.2015.05.018

**Published:** 2016-01-07

**Authors:** Debora Petrungaro Migueis, Luiz Claudio Santos Thuler, Lucas Neves de Andrade Lemes, Chirlene Santos Souza Moreira, Lucia Joffily, Maria Helena de Araujo-Melo

**Affiliations:** a Postgraduate Program in Neurology, Universidade Federal do Estado do Rio de Janeiro (UNIRIO), Rio de Janeiro, RJ, Brazil; b Clinical Investigation Division, Instituto Nacional de Câncer (INCA), Rio de Janeiro, RJ, Brazil; c Universidade do Estado do Rio de Janeiro (UERJ), Rio de Janeiro, RJ, Brazil; d Universidade Federal do Estado do Rio de Janeiro (UNIRIO), Rio de Janeiro, RJ, Brazil

**Keywords:** Nasal obstruction, Obstructive sleep apnea, Sleep fragmentation, Polysomnography, Treatment outcome, Obstrução nasal, Apneia obstrutiva do sono, Fragmentação do sono, Polissonografia, Resultado do tratamento

## Abstract

**Introduction:**

Obstructive sleep apnea syndrome (OSAS) is a common disorder that can lead to cardiovascular morbidity and mortality, as well as to metabolic, neurological, and behavioral consequences. It is currently believed that nasal obstruction compromises the quality of sleep when it results in breathing disorders and fragmentation of sleep. However, recent studies have failed to objectively associate sleep quality and nasal obstruction.

**Objective:**

The aim of this systematic review is to evaluate the influence of nasal obstruction on OSAS and polysomnographic indices associated with respiratory events.

**Methods:**

Eleven original articles published from 2003 to 2013 were selected, which addressed surgical and non-surgical treatment for nasal obstruction, performing polysomnography type 1 before and after the intervention.

**Results/conclusions:**

In most trials, nasal obstruction was not related to the apnea–hypopnea index (AHI), indicating no improvement in OSAS with reduction in nasal resistance. However, few researchers evaluated other polysomnography indices, such as the arousal index and rapid eye movement (REM) sleep percentage. These could change with nasal obstruction, since it is possible that the nasal obstruction does not completely block the upper airways, but can increase negative intrathoracic pressure, leading to sleep fragmentation.

## Resumo

**Introdução:**

A síndrome da apneia obstrutiva do sono (SAOS) é um distúrbio muito prevalente que pode ocasionar morbi-mortalidade cardiovascular, além de consequências metabólicas, neurológicas e comportamentais. Atualmente, acredita-se que a obstrução nasal comprometa a qualidade do sono, devido a distúrbios respiratórios e fragmentação do sono. Entretanto, até o momento estudos recentes não conseguem relacionar objetivamente qualidade do sono e obstrução nasal.

**Objetivo:**

O objetivo principal desta revisão sistemática é avaliar a influência da obstrução nasal na SAOS e em índices polissonográficos associados a eventos respiratórios.

**Método:**

Foram selecionados um total de 11 artigos originais de 2003 a 2013 com tratamentos cirúrgicos e não cirúrgicos da obstrução nasal, realizando a polissonografia do tipo 1 antes e após a intervenção.

**Resultados/conclusões:**

Na maioria dos ensaios, a obstrução nasal não se relacionou ao índice de apneia-hipopneia, indicando ausência de melhora da SAOS com a redução da resistência nasal. Entretanto, poucos pesquisadores avaliaram índices polissonográficos como o índice de despertares e o percentual do sono REM (movimento rápido dos olhos) que poderiam vir alterados, uma vez que a obstrução nasal possivelmente não obstrui completamente a via aérea superior, mas aumenta a pressão negativa intratorácica, levando à fragmentação do sono.

## Introduction

Obstructive sleep apnea syndrome (OSAS) is a very prevalent disorder, which may result in cardiovascular morbidity and mortality, as well as metabolic, neurological, and behavioral consequences. In the Brazilian population, this syndrome is a public health problem, affecting 32.8% of the population.^1^ OSAS is an anatomical and functional abnormality resulting from partial or total neuromuscular collapse of the upper airways (UA) during sleep, mainly with negative pressures during inspiration. This obstruction causes sleep fragmentation and intermittent hypoxia. The main areas of obstruction are the nose, palate, and tongue, but the obstruction may be multifactorial.^2^ Currently, it is believed that nasal obstruction impairs the quality of sleep in respiratory disorders, and also adversely affects the adoption and adherence to continuous positive airway pressure (CPAP), the gold standard for OSAS treatment.^3^ However, recent studies have failed to objectively associate the quality of sleep with nasal obstruction.^4^

According to the European Position Paper on Rhinosinusitis and Nasal Polyps (EPOS 2012), nasal obstruction can be caused by several types of chronic (CRS) or acute rhinosinusitis.^5^ Some studies suggest that sleep complaints in patients with CRS are common and can even affect their quality of life, but there is little information about this association.^6^ The last review on the subject, carried out in 2013 by Meen et al., showed that drug and surgical nasal interventions did not improve the apnea–hypopnea index (AHI), or OSAS, but improved subjective symptoms of the disorder, such as excessive daytime sleepiness and quality of life.^4^ This and other more recent systematic reviews, however, did not evaluate the arousal index, RERA (respiratory effort-related arousals), and the sleep-disordered breathing index.

The main objective of this systematic review was to evaluate the influence of nasal obstruction on OSAS and other polysomnography indices associated with respiratory events, over the last decade.

## Methods

Articles selected were prospective studies, consisting of controlled clinical trials, and cohort studies, in which patients underwent type 1 polysomnography (supervised by the technician in the sleep laboratory), performed as a complete overnight study before and after conservative or surgical interventions to improve nasal breathing during sleep. Two reviewers selected the relevant literature published between 2003 and 2013 from MEDLINE (BIREME and PubMed), in English or Portuguese languages, on the association between nasal obstruction and OSAS. Related articles and references were also included in this review. Only original studies with surgical and non-surgical treatment of nasal obstruction that performed type 1 polysomnography before and after the intervention were selected. The following were excluded: letters to the Editor, case series (with less than ten patients), review articles, basic research studies, and studies without intervention or without type 1 polysomnography performed throughout the entire night. Studies that included patients with neuropathy, heart disease, age <18 years, and multilevel surgery or other non-nasal surgeries at the same time were also excluded.

The assessed interventions were: use of medications (nasal decongestants and topical corticosteroids), nasal dilators, and nasal surgeries (rhinoplasty, septoplasty with or without turbinectomy, functional endoscopic sinus surgery). In this systematic review, treatment success was evaluated according to subjective improvement in nasal obstruction and/or improvement in nasal resistance. Additionally, polysomnography indices related to respiratory events and pre and post-intervention EEG indices were compared to assess the influence of nasal obstruction on OSAS.

The search in PubMed was carried out in August 2014 using the terms “Nasal Obstruction” [MeSH] AND “Sleep Apnea, Obstructive” [MeSH], resulting in 140 articles. In the Virtual Health Library, using the terms “nasal obstruction and Obstructive Sleep Apnea”, the authors obtained 613 articles. After a review of titles and abstracts, the first reviewer obtained 21 articles from PubMed and 33 from BIREME. After excluding articles that were repeated in both sources, 52 studies remained. After reviewing the titles and abstracts, the second reviewer selected 21 articles from PubMed and 42 from BIREME. After eliminating the repeated articles, 46 remained.

**Figure 1:**
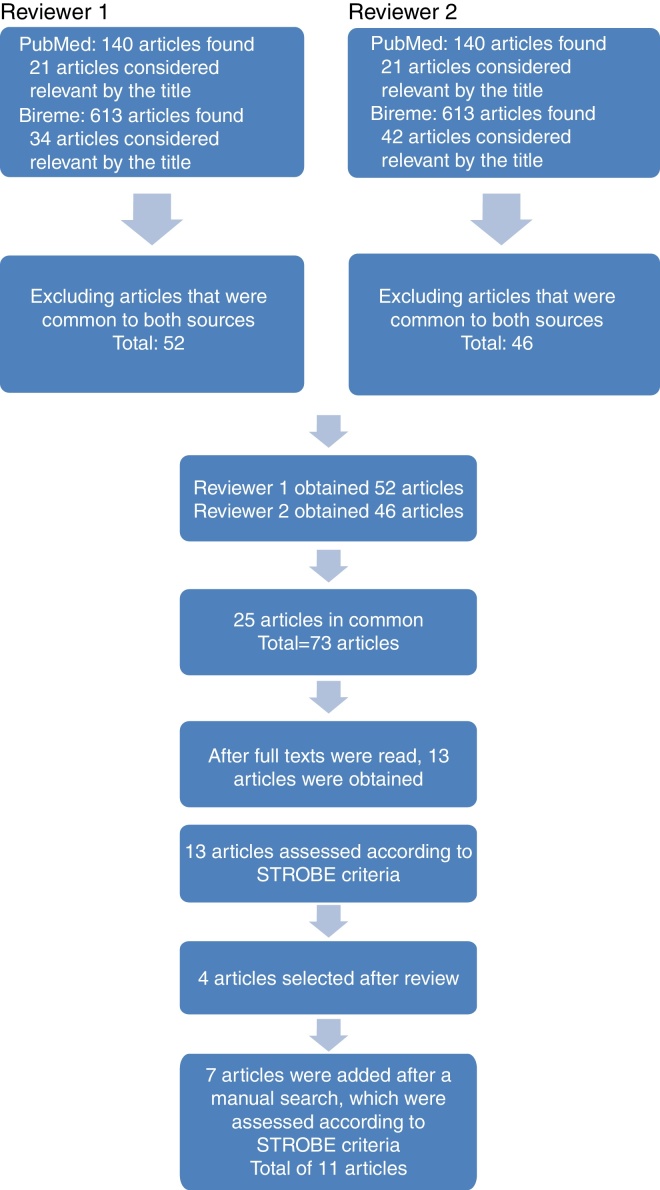
Literature review process. The articles were obtained by using the keywords in BIREME and PubMed. Each reviewer initially assessed 753 articles. After exclusion of articles repeated between sources, titles, and abstracts were evaluated together, which resulted in 73 articles that were assessed in full. There were 25 articles in common and 13 were evaluated according to the STROBE criteria. In addition to these, seven articles were included through manual search of the analyzed references.

Among the articles selected by both reviewers, 25 were repeated, and after assessing both the titles and abstracts, 73 articles remained to be read in full and finally selected. In addition to these, other articles were also included through manual search of the evaluated references (Fig. 1).

The level of significance was set at 5% (*α* = 0.05) to reject the null hypothesis. The values are shown with the respective 95% confidence intervals (95% CI), which expresses with 95% certainty the range of values within which the true value is found in the population.^7^ Median age and body mass index (BMI) were calculated as a central tendency measure. Additionally, all selected articles met the criteria established by Strengthening the Reporting of Observational Studies in Epidemiology (STROBE) applied to cohort studies.^8^

## Results

After selecting the full articles and evaluating the methodology, *p*-value, confidence interval, absence of bias, and the presence of all criteria established by the STROBE checklist, 11 articles were selected for this systematic review. Patients with nasal obstruction underwent clinical and surgical interventions to improve nasal breathing, comparing pre- and postoperative polysomnography indices. Excessive daytime sleepiness was assessed by the Epworth Sleepiness Scale (ESS)^9^ and clinical improvement.

The following polysomnographic parameters were evaluated: AHI, sleep-disordered breathing index (SDBI), presence of desaturation and snoring, arousal index, sleep architecture, REM (rapid eye movement) sleep, and slow-wave sleep (previously known as N3 + N4 sleep stage) according to the criteria of the American Academy of Sleep Medicine (AAMS) Manual.^10^

A total of 297 patients were evaluated, with a median age of 46 years and a mean BMI of 27.9 kg/m^2^.

Of the three trials with drug treatment (Table 1), all patients obtained a reduction in nasal resistance and improved subjective sleep quality, without changing the snoring. After conservative treatment, the AHI and the desaturation index only showed a significant reduction in study by Kiely et al. ^11^ Two trials using decongestants^12^, ^13^ demonstrated no improvement in excessive daytime sleepiness according to the Epworth Sleepiness Scale (ESS). In two studies with clinical interventions,^11^, ^12^ there was a significant increase in slow-wave sleep, and only Lean et al.^12^ found a lower arousal index, higher sleep efficiency, and increased percentage of REM sleep and slow-wave sleep (Table 2).

**Table 1:** Total number of studies with clinical and surgical intervention.

Authors and year of publication	Follow-up period	Study design	Patients (*n*)	Males (%)	Mean age	Mean BMI	Nasal intervention	AMMS manual
Kiely et al.^11^ 2004	2 months	Clinical trial	23	82.6	46	27.9	Fluticasone spray 100 mcg 2×/day for a month and placebo for a month. Crossover design.	1999
Lean et al.^12^ 2005	2 PSG with a one-day interval between them	Clinical trial	10	90	46.5	27	Nasal decongestant 1 h before lights-out and nasal dilator. Crossover design.	1999
Clarenbach et al.^13^ 2008	3 weeks	Clinical trial	12	83.3	49.1	30.7	Patients with EDS, OSAS, and nasal complaints in two randomized groups: one with topical xylometazoline and another with placebo for seven days. Crossover design.	1992
Nakata et al.^14^ 2005	PSG pre and post-op	Clinical trial	12	100	54.2	27	Inferior turbinectomy and septoplasty. Sinusotomy in one patient. CPAP use pre- and postoperatively.	1999
Virkkula et al.^15^ 2006	2–6 months	Prospective study	40	100	44.2	27.9	Septoplasty with (2) or without partial inferior turbinectomy and rhinoseptoplasty (two patients).	1999
Koutsourelakis et al.^16^ 2008	PSG pre and post-op	Clinical trial	49	75.5	38.3	30.15	27 septoplasties with (18) or without partial inferior turbinectomy, 22 sham surgeries.	1999
Li et al.^17^ 2008	3 months	Clinical trial	51	98	39	26	Septoplasty and sinusectomy.	1999
Tosun et al.^18^ 2009	3 months	Clinical trial	27	81.5	40.37	23.87	FESS in patients with sinonasal polyposis (obstruction ≥50% of each nasal passage).	1999
Bican et al.^19^ 2010	4 months	Prospective study	20	100	47.5	31	Rhinoseptoplasty, with emphasis on the nasal valve, improvement and CPAP pre and post-op.	1999
Choi et al.^20^ 2011	3 months	Prospective study	22	100	41.3	25.5	After the use of topical steroids without nasal obstruction improvement, they were submitted to nasal surgery (5 endoscopic, 17 septoplasties with turbinectomy).	2007
Sufioğlu et al.^21^ 2012	3 months	Prospective study	31	83.9	53	30.3	Surgeries: (1) three septoplasties, (2) two rhinoseptoplasties, (3) eighteen septoplasties and turbinectomies, (4) four sinusectomies, septoplasties and turbinectomies (5) four bilateral inferior turbinectomies.	2007

EDS, excessive daytime sleepiness; OSAS, obstructive sleep apnea syndrome; Pre-op, pre-operatively; Post-op, post-operatively; FESS, functional endoscopic sinus surgery; PSG, polysomnography; CPAP, continuous positive airway pressure.

**Table 2:** Changes with clinical treatment.

Authors and year	Nasal resistance	Snoring after the intervention	Clinical improvement	Polysomnography after intervention	AHI and SDBI	Arousal index
Kiely et al. 2004	Reduction^a^ with active treatment.	No reduction.	Improved daytime alert by the daily record and quality of sleep.	Limited effect in the treatment of OSAS.	AHI and desaturation index decreased^a^ with fluticasone.	Not reported.
				Increase^a^ of SWS.		
Lean et al. 2005	Reduction^a^ with active treatment.	Not reported.	Reduction^a^ of mouth breathing during sleep and partial improvement of sleep quality.	Improvement^a^ of sleep efficiency.	No reduction.	Reduction^a^ with active treatment.
			No reduction in ESS.	Increase^a^ in REM and SWS.		
Clarenbach et al. 2008	Reduction^a^ with active treatment.	No reduction.	No reduction in ESS.	No alteration in SWS or REM.	No reduction.	No reduction.

AHI, apnea and hypopnea index; SDBI, sleep-disordered breathing index; ESS, Epworth Sleepiness Scale; SWS, slow-wave sleep; CPAP, continuous positive airway pressure; desaturation index, number of desaturations ≥4% per hour of sleep.

a Statistically significant difference.

Among the eight studies with surgical intervention^14^, ^15^, ^16^, ^17^, ^18^, ^19^, ^20^, ^21^ (Table 1), all achieved significant reduction in nasal resistance. Only one trial with surgical intervention^15^ did not achieve significant change in the ESS, while the others showed a reduction in excessive daytime sleepiness. However, after the intervention, only two that used CPAP (Bican et al. ^19^ and Sufioğlu et al. ^21^) showed significant reduction in AHI and CPAP pressure. After surgery and the use of CPAP, Nakata et al.^14^ showed decrease in CPAP pressure, without reduction in the AHI.

Four studies^17^, ^18^, ^20^, ^21^ showed a reduction of snoring, and Sufioğlu et al.^21^ reported that this improvement was subjective. Only two studies^14^, ^19^ showed increase in the minimum nocturnal oxygen saturation postoperatively. In addition, Bican et al.^19^ and Choi et al.^20^ showed an increase in total sleep time and increase in the percentage of REM sleep. Only one study^21^ showed an increase in N3 + N4 sleep (slow-wave sleep). No study with surgical intervention assessed or demonstrated any changes in the arousal index (Table 3).

**Table 3:** Changes with surgical treatment.

Authors and year	Nasal resistance	Snoring after intervention	Clinical improvement	Polysomnography after intervention	Arousal index	AHI and SDBI
Nakata S 2005	Reduction^a^	Not reported.	Reduction^a^ in ESS.	CPAP pressure reduction in 5 patients.	Not reported.	Did not change AHI with CPAP pre and post-op.
			Better adaptation to CPAP.	Increase^a^ in the minimum oxygen saturation.		
Virkkula P 2006	Reduction^a^	No reduction^a^.	No improvement in nocturnal breathing and in ESS post-op.	No reduction in the desaturation index, arousals and duration of snoring in individuals with normal cephalometry or not.	No change.	Did not change AHI in individuals with normal cephalometry or not.
Koutsourel akis I 2008	Reduction^a^	Not reported.	Reduction^a^ in the ESS after nasal surgery, different from placebo.	Not informed	Not reported.	Did not change the AHI with nasal surgery or placebo.
Li HY 2008	Reduction^a^	Snoring decreased^a^.	Improved^a^ nasal breathing at the visual analog scale of nasal obstruction in 98% of patients.	No changes in the minimum oxygen saturation three months post-op.	Not reported.	No change.
			Subjective^a^ sleep improvement.			
			Reduction^a^ in ESS.			
Tosun F 2009	Reduction^a^	Snoring decreased^a^ in all patients and disappeared completely in 9 of the 27.	Reduction^a^ in ESS.	Improved^a^ quality of sleep.	No change.	No change.
				No changes in the minimum oxygen saturation in post-op.		
Bican A 2010	Reduction^a^	Not reported	Reduction^a^ in ESS in post-op of patients with CPAP.	Increase^a^ in REM.	Not reported.	AHI decreased^a^.
				Increase^a^ in N1, N2 and total sleep time, in the post-op.		Reduction^a^ in pressure to CPAP in the post-op.
				No difference in N3 + N4 sleep.		
			Improved^a^ subjective comfort of nasal flow.	Increase^a^ in the minimum oxygen saturation.		
Choi JH 2011	Reduction^a^	Snoring decreased^a^.	Reduction^a^ in ESS.	Increase^a^ in REM.	No change.	Did not change the AHI or the minimum oxygen saturation, with isolated nasal surgery.
				Increase^a^ in total sleep time and sleep efficiency.		
Sufioğlu M 2012	Reduction^a^	Subjective improvement^a^ only of snoring.	Reduction^a^ in ESS.	Increase^a^ in N3 + N4.	Not reported.	Did not change the AHI. The AHI decreased to less than 5/h in 5 patients, which means the cure of OSAS.
			Increase^a^ in CPAP tolerance.			
			Improvement^a^ of subjective complaints of obstruction, snoring, apnea and daytime sleepiness.	Reduction of pressure of CPAP in the post-op.		Reduction* of total duration of apneas and hypopneas.

AHI, apnea and hypopnea index; SDBI, sleep-disordered breathing index; ESS, Epworth Sleepiness Scale; TST, total sleep time; N3 + N4, slow-wave sleep; CPAP, continuous positive airway pressure.

a Statistically significant difference (*p* < 0.05).

## Discussion

Sleep-disordered breathing (SDB), according to the Third International Classification of Sleep Disorders (ICSD-3),^22^ is characterized by ventilation abnormalities during sleep and, sometimes may be present during wakefulness. It comprises four categories: OSAS, central sleep apnea, sleep-related hypoventilation/hypoxemia, and upper-airway resistance syndrome (UARS); individuals can display more than one condition. This review shows a series of 297 cases, in which patients with different causes of nasal obstruction were submitted to clinical and surgical interventions, and were evaluated for polysomnography indices and clinical improvement.

OSAS was the best-studied and most accepted disorder in the medical community. It is characterized by partial or total obstruction of the upper airways, called hypopnea and apnea, with episodic drops in oxyhemoglobin saturation and recurrent awakenings.^10^ In addition to these events, respiratory effort-related arousals (RERA) may occur, without apnea or hypopnea, maintaining oxyhemoglobin levels stable during sleep. These awakenings have consequences, such as sleep fragmentation and excessive daytime sleepiness, and are related to another SDB known as UARS.^23^, ^24^ Only Sufioğlu et al.^21^ assessed sleep fragmentation, demonstrating the effects on sleep architecture, showing the scarcity of studies about this aspect.

Nasal medications did not improve snoring. Two studies^12^, ^13^ used vasoconstrictors for a short period, but both only reduced nasal resistance and improved subjective aspects of sleep. Possibly, the chronic use of vasoconstrictors might not have the same effect, as it could result in drug-induced rhinitis.

All studies with surgical intervention^14^, ^15^, ^16^, ^17^, ^18^, ^19^, ^20^, ^21^ decreased nasal resistance, with most of them resulting in the reduction of snoring and excessive daytime sleepiness, although they did not reduce AHI. Two studies^19^, ^21^ showed significant reduction in AHI. Sufioğlu et al.^21^ demonstrated the increase in the slow-wave sleep percentage. Two trials^19^, ^20^ showed an increase in total sleep time and percentage of REM sleep. In some studies, the sleep architecture was not reported, indicating the need for better study of this aspect with significant behavioral and neurological effects. No surgical intervention evaluated or showed any change in the arousal index. An increase of this index suggests airflow limitation that causes micro-arousals, with consequent sleep fragmentation and sometimes, intermittent hypoxia. This not only would result in metabolic disorders, but also irritability, anxiety, difficulty in consolidating memory, and reduced concentration and attention, which could impair the individual's productivity.^23^, ^24^

Three studies that used CPAP^14^, ^19^, ^21^ showed that it was possible to reduce the pressure necessary for effective use following intervention, which improved treatment adherence. Only Nakata et al.^14^ and Bican et al.^19^ showed increase in the minimum oxygen saturation after surgery, which can result in metabolic and neurological benefits to the individual.

This review showed that many authors consider the AHI to be very important, without assessing the arousal index and sleep architecture. This may result in the underdiagnosis of the UARS, impairing the understanding of excessive sleepiness associated with it, which could deprive patients of a treatment that could bring them benefits.

Only two studies, carried out in 2011 and 2012, used the 2007 AAMS Manual, indicating that the others did not evaluate RERA and the SDBI, the sum of the number of apneas, hypopneas, and RERA divided by total sleep time. In the last task force to prepare the 2012 AAMS Manual, RERA measurement became mandatory, an airflow limitation with the formation of a plateau in the nasal cannula, lasting 10 s, associated with awakening. In the 2007 AAMS Manual, measuring the number of RERA was optional, despite the relevance of UARS and SDBI.

Recently, arousals have been studied more frequently. Terzano et al.^25^ described arousals with a cyclic alternating pattern (CAP) during non-REM (NREM) sleep in patients with normal AHI, but high rate of respiratory disorders. They had UARS with fatigue and daytime sleepiness, despite normal AHI, reinforcing the association between the number of CAP, indicative of NREM sleep fragmentation, with the Epworth Sleepiness Scale. However, the CAP has not been established as a criterion in the AMMS-2012, indicating the need for further studies to reinforce its clinical significance. Finally, the inclusion of CAP has altered some paradigms.

Arousal is currently defined as frequencies greater than 16 Hz (no zones), preceded by 10 s of sleep, lasting more than 3 s, while CAP lasts longer than 2 s. The inclusion, for instance, of the CAP in AAMS Manual can increase the sensitivity of the polysomnography study, allowing the diagnosis, treatment, and monitoring of previously neglected disorders. The standard polysomnographic report of most studies in this review does not allow the quantification of aspects with significant clinical repercussions.

The articles by Choi et al.^20^ and Sufioğlu et al.^21^ from 2011 and 2012, respectively, used the AMMS-2007 Manual, commenting on sleep fragmentation and arousal index.

Friedman et al.,^26^ showed that patients with moderate to severe OSAS who underwent nasal reconstruction, postoperatively exhibited worse objective sleep study findings. Possibly, this was due to an existing neuromuscular change in the upper airway that was not corrected through an intervention exclusively performed at the nasal level. Indeed, during muscle relaxation, patients with less fragmented sleep can have more REM sleep, as well as more apnea and hypopnea. However, this paradoxical effect of nasal surgery on the SDBI requires further study.

One factor that complicates the definition of therapeutic success is the lack of parameters for OSAS improvement. One of the most commonly used criterion for intervention success is an improvement of SDBI to ≤50% of the preoperative value, with a preoperative value of <20 events per hour.^27^ However, there are criticisms regarding its use for severe OSAS or in patients with pre-intervention SDBI values close to 20 events per hour. Other success criteria were created, such as a reduction in the SDBI to less than five events per hour, improvement in oxygen saturation to levels >90%, and significant reduction of events,^28^ but these do not adequately assess the improvement of patients with severe OSAS. Thus, a consensus regarding this definition is needed.

Another aspect observed during the selection of articles was the increasing number of studies performed with portable polysomnography without the presence of a technician (polysomnography type 2). These articles were excluded from this review. The AMMS-2012 Manual^10^ and ICSD-3^22^ consider portable polysomnography a useful tool in clinical practice, but the possible loss of the quality of the examination due to lack of supervision by a technician should be even better established by research.

## Conclusion

We observed a large number of clinical trials that used septal deviation and allergic rhinitis as factors in nasal obstruction during the last ten years. Only one study considered nasal polyposis (NP) as a cause of obstruction. Persistent allergic rhinitis is an important factor of nasal obstruction, but its intensity may vary. NP has more objective tools for assessing the severity of the obstruction.

Only four studies recorded a significant improvement in snoring; three studies showed a reduction in CPAP pressure and seven reported subjective sleep improvement. Thus, the nasal role on the physiopathology of OSAS remains imprecise. Reduction in excessive daytime sleepiness was observed in some studies, measured by the Epworth Sleepiness Scale.

In most trials, nasal obstruction was not associated with AHI, indicating no improvement in OSAS with nasal resistance reduction. In contrast, few researchers evaluated polysomnography indices, such as the arousal index and percentage of REM sleep, which could be altered, as nasal obstruction sometimes does not cause complete upper airway obstruction, but increases the negative intrathoracic pressure, leading to sleep fragmentation. Thus, more studies are required on the influence of nasal obstruction on polysomnography.

## Conflicts of interest

The authors declare no conflicts of interest.

## References

[1] Tufik S., Santos-Silva R., Taddei J.A., Bittencourt L.R. (2010). Obstructive sleep apnea syndrome in the Sao Paulo Epidemiologic Sleep Study. Sleep Med.

[2] Pang K. (2013). The role of nasal surgery in the treatment of OSA. Curr Otorhinolaryngol Rep.

[3] Poirier J., George C., Rotenberg B. (2014). The effect of nasal surgery on nasal continuous positive airway pressure compliance. Laryngoscope.

[4] Meen E.K., Chandra R.K. (2013). The role of the nose in sleep-disordered breathing. Am J Rhinol Allergy.

[5] Fokkens W.J., Lund V.J., Mullol J., Bachert C., Alobid I., Baroody F. (2012). EPOS 2012: European position paper on rhinosinusitis and nasal polyps 2012. A summary for otorhinolaryngologists. Rhinology.

[6] Alt J., Smith T., Mace J., Soler Z. (2013). Sleep quality and disease severity in patients with chronic rhinosinusitis. Laryngoscope.

[7] Medronho R.A., Bloch K.V. (2008).

[8] Von Elm E. (2014). http://www.strobe-statement.org/pdf/index.php?id=available-checklists.

[9] Johns M.W. (1991). A new method for measuring daytime sleepiness: the Epworth sleepiness scale. Sleep.

[10] Berry R.B., Brooks R., Gamaldo C.E., Harding S.M., LIoyd R.M., Marcus C.L., for the American Academy of Sleep Medicine (2012).

[11] Kiely J.L., Nolan P., McNicholas W.T. (2004). Intranasal corticosteroid therapy for obstructive sleep apnoea in patients with co-existing rhinitis. Thorax.

[12] McLean H., Urton A., Driver H., Tan A.K., Day A.G., Munt P.W. (2005). Effect of treating severe nasal obstruction on the severity of obstructive sleep apnoea. Eur Respir J.

[13] Clarenbach C.F., Kohler M., Senn O., Thuenheer R., Bloch K. (2008). Does nasal decongestion improve obstructive sleep apnea?. J Sleep Res.

[14] Nakata S., Noda A., Yagi H., Yanagi E., Mimura T., Okada T. (2005). Nasal resistance for determinant factor of nasal surgery in CPAP failure patients with obstructive sleep apnea syndrome. Rhinology.

[15] Virkkula P., Bachour A., Hytönen M., Salmi T., Malmberg H., Hurmerinta K. (2006). Snoring is not relieved by nasal surgery despite improvement in nasal resistance. Chest.

[16] Koutsourelakis I., Georgoulopoulos G., Perraki E., Vagiakis E., Roussos C., Zakynthinos S.G. (2008). Randomised trial of nasal surgery for fixed nasal obstruction in obstructive sleep apnoea. Eur Respir J.

[17] Li H.Y., Lin Y., Chen N.H., Lee L.A., Fang T.J., Wang P.C. (2008). Improvement in quality of life after nasal surgery alone for patients with obstructive sleep apnea and nasal obstruction. Arch Otolaryngol Head Neck Surg.

[18] Tosun F., Kemikli K., Yetkin S., Ozgen F., Durmaz A., Gerek M. (2009). Impact of endoscopic sinus surgery on sleep quality in patients with chronic nasal obstruction due to nasal polyposis. J Craniofac Surg.

[19] Bican A., Kahraman A., Bora I., Kahveci R., Hakyemez B. (2010). What is the efficacy of nasal surgery in patients with obstructive sleep apnea syndrome?. J Craniofac Surg.

[20] Choi J.H., Kim E.J., Kim Y.S., Kim T.H., Choi J., Kwon S.Y. (2011). Effectiveness of nasal surgery alone on sleep quality, architecture, position, and sleep-disordered breathing in obstructive sleep apnea syndrome with nasal obstruction. Am J Rhinol Allergy.

[21] Sufioğlu M., Ozmen O.A., Kasapoglu F., Demir U.L., Ursavas A., Erişen L. (2012). The efficacy of nasal surgery in obstructive sleep apnea syndrome: a prospective clinical study. Eur Arch Otorhinolaryngol.

[22] American Academy of Sleep Medicine (2014).

[23] Guilleminault C., Stoohs R., Clerk A., Cetel M., Maistros P. (1993). A cause of excessive daytime sleepiness. The upper airway resistance syndrome. Chest.

[24] Palombini L., Lopes M.C., Tufick S., Guilleminault C., Bittencourt L.R. (2011). Upper airway resistance syndrome: still not recognized and not treated. Sleep Sci.

[25] Terzano M.G., Parrino L., Smerieri A., Chervin R., Chokroverty S., Guilleminault C. (2002). Atlas, rules, and recording techniques for the scoring of cyclic alternating pattern (CAP) in human sleep. Sleep Med.

[26] Friedman M., Tanyeri H., Lim J.W., Landsberg R., Vaidyanathan K., Caldarelli D. (2000). Effect of improved nasal breathing on obstructive sleep apnea. Otolaryngol Head Neck Surg.

[27] Friedman M., Ibrahim H., Bass L. (2002). Clinical staging for sleep-disordered breathing. Otolaryngol Head Neck Surg.

[28] Ephros H.D., Madani M., Yalamanchili S.C. (2010). Surgical treatment of snoring & obstructive sleep apnoea. Indian J Med Res.

